# Waiting for the Doctor: Managing Time and Emotion in the British National Health Service, 1948–80

**DOI:** 10.1093/tcbh/hwab040

**Published:** 2021-12-14

**Authors:** Martin D Moore

**Affiliations:** University of Exeter, UK

## Abstract

This article examines patients’ and doctors’ emotional and psychological entanglements with the development of appointment systems in British general practice between the 1948 and 1980. Waiting, especially in the form of the queue, has been subject to recent historical analyses. However, the focus has often been on negative emotional responses, on how waiting has been politicized, and on the disciplinary power of the waiting room. Frameworks of rationalization and discipline have also dominated historical and sociological assessments of temporal regulation, and especially the rise of standardized, ordered, clock and calendar time that appointments embodied. Though productive, focusing too closely on these processes in relation to time and waiting risks underplaying the complex affective life of regulatory technologies, for both their operators and their subjects. By focusing tightly on how myriad, often contradictory, responses to appointment systems operated within the setting of post-war general practice, this article looks to place such emotional and psychological relations in historical context. In so doing, it develops recent work on the emotional history of the National Health Service and, by extension, of the diverse affective and temporal modes of the British welfare state.

In 1949, a Mass Observation researcher arrived at a general practitioner’s (GP) surgery, run from the doctor’s home in a ‘north-western suburb of London, near a large borough council estate and an extensive working-class shopping centre’. Although doors opened at quarter-to-nine in the morning, three women were already waiting outside at half-past-eight. Once inside, a bottleneck built. The GP ‘refuses to be rushed’ in consultation, with each patient seen in the order of their arrival. ‘Positions are worked out right from the beginning’ as ‘everyone adheres unquestioningly’ to ‘the discipline of the queue’. A full waiting room provides abundant opportunities for patients to interact, though ‘the place fixes a pattern’ on conversation: doctors and diseases take centre-stage. Eventually, the two-hour wait for some patients erodes any discussion. ‘Long gaps of yawning silence’ are broken only by complaints about unnecessary attendances and the doctor’s speed, and, ultimately, by an invitation to enter the consulting room.^1^

This scene, with its domestic setting, improvised queuing and potential lengthy wait, would have been familiar to many readers in the late 1940s. However, despite its persistence in popular culture, such experiences of waiting were increasing uncommon 30 years later. On the one hand, most GPs had moved into new, purpose-built accommodation by the 1980s.^2^ On the other, waiting room attendance was ever-more regulated by comprehensive appointment systems: whereas around 2 per cent of GPs operated such systems in 1950, estimates for the mid-1970s were closer to 80 per cent.^3^

The introduction of full-time appointment systems for surgery sessions significantly disrupted how patients and doctors experienced time and waiting in British general practice at the level of the ‘everyday’.^4^ Patients with appointments, in Laura Salisbury’s terms, generally passed less time collectively waiting *with* one another in the waiting room, whilst enforced waiting *for* an appointment outside the surgery became more common.^5^ Appointments decoupled attendance by the doctor from the time of a patient’s arrival at surgery, and consultations would, at least ostensibly, be bracketed by the clock rather than the waiting-room crowd. Similarly, for doctors, the ‘free-for-all’ of open surgery sessions was now given structure, moving at a pace set by GPs rather than the weight of demand.^6^ Of course, systems broke down, and a minority of GPs preferred what they saw as an ‘open house’ policy.^7^ Nonetheless, by the early 1980s, transformations in the ‘temporal architecture’ of general practice had subjected time and waiting in the practice to a new order.^8^

From a certain perspective, GPs’ deployment of appointment systems fits neatly within functionalist analyses of time-discipline, synchronization and rationalization.^9^ With the arrival of the National Health Service (NHS), it might be argued that a previously domestic, individualistic general practice was integrated into a ‘modern’ mass organization, akin to the factory or large-scale bureaucracy.^10^ Although not the case for all GPs, the NHS’s universal coverage undoubtedly created new pressures and increased workloads. In this framework, appointment systems could be seen as simple productivity tools, eliminating ‘task-oriented’ forms of work and reducing time to the empty, calculable, fungible unit of modernity.^11^ To enhance efficiency, appointments thus synchronized the personal time of both practitioners (as workers) and patients (as objects) with the standardized clock and calendar of the institution, enforcing new forms of temporal discipline on all.^12^ Patients’ waiting now emerged from the ‘redundancies’ of these standardized ‘time regimes’, and reflected the priority of medical time within a comprehensive health service.^13^

While the incorporation of general practice into modern structures of healthcare provides important context for the transformation of time and waiting, holding too fast to a rationalization framework risks erasing the complex affective life of appointment systems and the temporal regulation they helped inaugurate.^14^ The introduction of appointment systems was far from an autonomic, unsentimental response to organizational need. Although calculated and ordered in standardized minutes, hours and working days, appointments were temporal promises of attention to people in states of illness and uncertainty. Equally, they not only reworked long-established social practices and cultural expectations of waiting and care, they also emerged as a response to their stresses and strains. Appointments, and the times they reordered, thus generated deep and varied emotional responses and investments. Some patients became attached to appointment systems, particularly the freedom they offered to organize life around competing demands; others nostalgically lamented a lost personal contact with, and availability of, the doctor. Likewise, whilst many GPs appreciated how appointments alleviated the stressful rush and interminable duration of open surgeries, a minority worried how new temporal regulations would negatively affect patients. Along with lay commentators, they expressed anxieties about the dangers of ‘modern’ general practice.

This article, then, re-examines early debates about, and encounters with, appointment systems in general practice, using discussions prompted by their emergence to articulate how appointments were intricately intertwined with emotional and psychological life. It argues that waiting and temporal ordering were deeply affective practices—bound up with irritation, boredom, anxiety, guilt, relief, and pleasure—but that such experiences were themselves shaped by shifting post-war social structures, cultural expectations, institutional arrangements and political narratives. Questions of status, gender roles, race, professional ambition and duty, community dislocation, and discourses of modernization pervaded post-war Britain and its welfare state, and all impinged on what it meant to wait for the doctor.^15^ To paraphrase Ghassan Hage, the following analysis thus underlines how waiting and temporal regulation are always simultaneously existential and historical phenomena.^16^

Moreover, in making these arguments, this article combines and develops two nascent bodies of literature. First, it furthers recent historical scholarship on queuing and medical waiting in the twentieth century, in which issues of discipline, management and politicization have been central.^17^ Where this work has explored emotional responses to waiting, the focus has tended towards waiting’s irritations and horrors rather than its rich variety of affective experiences.^18^ Equally, whilst Joe Moran’s pioneering work has provided an important analysis of the shifting social and symbolic life of the post-war queue, his perspective has also been panoramic.^19^ By focusing on historical transformations within one setting—the general practice surgery—this article is able to examine the interplay of broader cultural and political change with the *particular* expectations and routines of a given context in shaping experiences of waiting and temporal regulation.

Secondly, this article contributes to an emergent historiography on emotional histories of the NHS and post-war welfare state. Recent work has examined the ‘emotional landscapes’ of post-war surgery, trade unionism, and the affective life of the welfare state’s embedded racism, sexism and colonial inheritance.^20^ Jennifer Crane, Hannah Elizabeth and others have also explored how specific affective states became objectified, suppressed and mobilized in strategies of governance and political struggles over healthcare.^21^ However, whilst this literature has generated important insights onto the affective experiences of healthcare workers and the emotional communities of policy-makers, it has not placed medical professionals and patients together in the same frame. It has not, to paraphrase Joanna Bourke, examined what the emotional entanglements of NHS professionals and patients *did*.^22^

Indeed, this literature has rarely engaged explicitly with the history of emotions or critical orientations to affect theory.^23^ This article, therefore, looks to follow the lead set by Stephen Brooke and Hannah Elizabeth, to show how a more sustained exploration of the health and welfare services using critical historical approaches to emotion can open up new perspectives on NHS history.^24^ To do so, it turns not to foundational figures in histories of emotion, such as William Reddy, Barbara Rosenwein, or Peter and Carol Stearns, nor to intellectual historical and constructionist literature around psychological and emotional states.^25^ Such work has proven useful for thinking through how historical conditions and welfare institutions produced new framings and experiences of the self, and structured the expression of feelings and emotional codes. However, this article is concerned neither with the creation of new emotional states, nor strictly about the performance or governance of emotion. Rather, it focuses on out how diverse affective experiences were a core feature of how people experienced and engaged with general practice under the NHS. And it draws on Sara Ahmed (and Brooke’s own historical mobilization of Ahmed’s work) to consider how emotions, as historically-contingent and historically-bound affective states, simultaneously informed social action and circulated around certain figures and temporal objects.^26^

Combining these bodies of scholarship, therefore, this article shows that the intense ‘affective ecologies’ that developed around time and waiting in post-war general practice—the site of the vast majority of post-war clinical encounters between healthcare professionals and patients—had profound implications for the everyday work of the wider NHS.^27^ Situating GPs’ and patients’ affective experiences of time within particular socio-historical conditions shows the ways these feelings shaped how different patients and doctors experienced care, saw one another, and operated in—and interacted with—a major institution of the post-war welfare state. The positionality of different actors relative to both the delivery of care and other social and cultural hierarchies meant that particular feelings became associated with, or ‘stuck to’, certain figurative and material bodies more than others.^28^ Classism and sexism, for instance, structured resentment around female receptionists’ ‘officiousness’. However, it is only by taking affective investment seriously that we can understand how particular structures of time came to exist, persist and be subjected to either intense criticism or a staunch defence.^29^

To make its case, this article begins with a brief history of open surgery sessions in general practice, and articulates the contrasting experiences of time such encounters generated for post-war patients and doctors. Using this as a point of comparison, section two explores how and why GPs turned to full-time appointments systems, and how patients responded—affectively and socially—to their new temporal management. The final section considers how feelings of frustration, anxiety and nostalgia circulated around the figures of the ‘family doctor’ and ‘receptionist’ after 1948. Though small in numbers, vocal critics associated appointment systems, and the increasing visibility of reception staff, with a loss of traditional family doctoring, and their critiques intertwined with broader anxieties about ‘modernisation’. They thus underline how responses to appointment systems were intertwined with a wider history of post-war social and cultural change, and how matters of temporal organization altered patients’ and practitioners’ affective relations to the NHS itself.

## General Practice and the Open Surgery Session

To understand why GPs deployed appointment systems, and how appointments assumed particular meanings after 1948, it is first necessary to see how doctors and patients felt about the appointment’s predecessor: the ‘open’ surgery session. Appointment systems appealed to some doctors precisely because they regulated the overwhelming demands of open surgeries—hours at which any registered patient could attend for consultation, and during which they were entitled to attention provided they arrived before doors closed. In contrast, critics’ personal and cultural memories of such disorganized time positioned open surgeries as more personal than the over-regimented ‘inhumanity’ of appointments. The open surgery, therefore, acted as an emotional and temporal shadow to appointment systems, making it impossible to understand one without the other.

Open surgery hours date back to at least the late-nineteenth century. As Anne Digby has noted, doctors seeking new markets for their services used fixed hours at surgery premises to attract patients.^30^ Similarly, doctors contracted to friendly societies and workers’ clubs used fixed sessions to reduce time-costly home visits and improve throughput.^31^ As club practice paid a fixed fee per patient rather than per consultation, time compression freed time for other activities.^32^ The introduction of state-mandated National Health Insurance (NHI) in 1911, which used a similar capitation fee, institutionalized the practice. NHI did not cover the whole population, extending only to employed persons aged between 16 and 70 years (thresholds later lowered) who earned under a certain income. Married women and children not in formal employment, as well as unemployed workers and older ‘dependents’, were excluded.^33^ Similarly, middle-class private patients might still insist on home visits or *ad hoc* appointments outside surgery hours.^34^ Nonetheless, NHI extended GP access considerably and, as most practitioners performed some NHI work, surgery hours became common to interwar practice for the same reasons as under club work.^35^ They became entrenched as the NHS removed insurance qualifications, and universal healthcare created greater pressure on medical time.

Patients’ accounts of waiting in open surgeries before and after 1948 are rare. However, two contrasting versions of surgery waiting dominated popular representations. Most common was a vision of surgery waiting as drab, depressing and interminable. Recalling a visit to their doctor in 1955, one *Daily Mail* columnist described patients seated along the wall of the waiting room ‘in a terrible silence as though smitten dumb by Providence’. This ‘trance-like condition’ was supposedly the intended effect of waiting room design: uninteresting magazines, uncomfortably arranged seating, and nonsensical signs were all deployed to break ‘the patient’s spirit’ and prevent them from active participation in the consultation.^36^ Two decades later, an *Observer* satirist offered similar tropes. His fellow ‘sufferers’ were ‘sunk in misery’, as the waiting room reduced the author to ‘*delerium tremens*’. Worse, however, was the tedium of waiting’s duration:

Remember ‘Waiting for Lefty’, Clifford Odet’s first play? … Well, then, how about ‘Waiting for Godot, by Samuel Beckett? … Lefty never got there. Neither did Godot. You have thoughts like these while waiting for a National Health Service doctor, which is not quite so long as waiting for a dead man (Lefty had contracted a terminal illness from bullets) or God, but only about 20 minutes shorter.^37^

Although undoubtedly exaggerated for comic effect, such accounts offer glimpses into the experiences and psychological states of waiting NHS patients. Whether apocryphal or not, their publication presumed some resonance with their readers.

However, a second, less common, representation of surgery waiting described it as a time when people might meet, interact, even fall in love.^38^ GPs contributed to this image of relationality with condescending descriptions of surgery sessions as ‘a social evening out’ for some patients, or as opportunities for ‘a few middle-aged women’ and ‘a few others’ to have a ‘weekly “gossip”’.^39^ Such comments undoubtedly demonstrated the contempt that many GPs had for patients they regarded as attending for ‘trivial’ reasons, and reproduced the sexism prominent in post-war medicine: discrimination in recruitment and employment ensured only around 6 per cent of senior GPs were women in the mid-1950s, and only 12 per cent of GPs were women by 1970.^40^ Nonetheless, suggestions that patients interacted whilst waiting aligned with accounts from patients and Mass Observers in the years after 1948.^41^

For patients, conversation may have been pleasurable socialization, but it also helped to manage the varied affective experiences of waiting. Read in light of Harold Schweizer’s work, for instance, it might be grouped with efforts at distraction—like reading or fidgeting—as a means to avoid existential musings that waiting could invoke, particularly in the aftermath of the Blitz and amid the Cold War.^42^ Alternatively, interaction could have been a conscious effort to alleviate what one patient-consumer magazine referred to in 1964 as a ‘boring, time and patience consuming situation’.^43^ Feelings of boredom and frustration had been raised by a Mass Observation investigation in 1949, which described patients complaining of being ‘unable to waste so much time’ as they had ‘so much to do at home’.^44^ Sociological research in the 1970s, moreover, found that patients’ anticipation of their consultation was often tinged by ‘fear’ (for instance of serious illness), ‘nervousness’, or ‘apprehension’ (about how the consultation might unfold ‘as a social activity’).^45^ Concerns about patients’ emotional states—their ‘feeling low, perhaps frightened’—even influenced post-war redesigns of GP premises, with emphasis on bright, cheerful and comfortable décor.^46^ Conversation, therefore, might have enabled patients to find pleasure, to contain or divert their feelings, or simply to exert some agency over their waiting whilst offering caring attention to those they waited *with*.^47^

In this regard, waiting in the practice surgery may not have been so different to queuing in other situations, at least into the 1950s. Queuers, especially in the ration queue, also reported experiencing feelings of boredom and frustration, and they noted the friendliness, complaints, conversation and ‘entertainers’ among fellow waiters with a mixture of appreciation and disdain.^48^ Equally, though the surgery wait did not descend into the ‘disordered shambles’ sometimes witnessed at the bus stop, the ire and murderous looks directed to the dishonest, selfish ‘queue jumper’ was common to many forms of waiting.^49^

In other respects, however, the surgery wait was a distinct experience. First, patients didn’t physically queue in the same way. Waiting rooms, at least into the 1960s, were generally adapted spaces (such as dining rooms or shop fronts), and patients in working-class industrial districts often complained of serious deficiencies in space, heating, and ventilation by ‘icy drafts’.^50^ Regardless, the provision of seating and a demarcated waiting space distinguished the surgery from the queue, and generally alleviated some of the physical exertion of standing. Secondly, unlike queuers for the bus or shop, patients attending open surgeries had a right to be seen. Unless there was an emergency, that is, there would be no waiting for long periods only to be told the object of waiting—the doctor—was no longer available.^51^

Finally, the doctor rarely drew the same anger and disgust as other figures identified as causes for delay. As Joe Moran has noted, wartime and post-war suggestions that queuing marked a peculiarly English sense of decency, democracy and fairness were quickly challenged by those subjected to queuing.^52^ On the one hand, vernacular critiques of fairness emerged among those forced to wait. ‘It is’, noted one Mass Observer in 1948, ‘very rarely that the people that need most what is in short supply, such as young mothers and invalids, can afford the time to queue’.^53^ In contrast, questioning the rights of other passengers, one *Daily Mail* (1955) reader suggested that there ‘should be priority tickets for workers’.^54^ On the other hand, stoked particularly by Conservative politicians and commentators, significant numbers of middle-class Britons came to query the necessity of queuing for certain goods into the 1950s; the bureaucratic shop-keeper and incompetent government official became targeted as obstacles by both frustrated queuers and new organizations, such as the British Housewives’ League.^55^ Yet, despite isolated press complaints about doctors ‘prefer[ring] the waiting room queue’, the GP largely avoided their waiting patients’ censure into the 1960s—whether out of respect, familiarity, or a reliance on GP’s expertise and gatekeeping power.^56^ Instead, as noted below, in later years, patients’ irritations over waiting would be directed towards a new figure in the surgery: the receptionist.

In contrast to their patients, GPs often experienced open surgeries as overwhelming tides of physical, mental and emotional work. For a minority of commentators, GPs’ experiences of overwork were framed expressly within older frameworks of industrial fatigue and organizational reform.^57^ More broadly, and indicative of their growing cultural and scientific significance, languages of stress and strain were mobilized by GPs to describe their surgery hours.^58^ Doctors in one 1960s’ practice described feeling like they were ‘working against time, knowing that 10 or more patients are waiting’ prior to establishing their appointment system. Under new arrangements, in contrast, there was ‘much less rush and stress’ as the doctors knew they were ‘keeping pace with our appointments’.^59^ Surveys similarly recalled how appointments could ‘relieve the doctor of the strain which a crowded waiting room puts upon him’.^60^ However, waiting patients provoked annoyance, anxiety and guilt, as well as stress. ‘For the family doctor’, wrote one GP, ‘a crowded waiting room is a source of irritation, as are surgeries of interminable length’ that resulted.^61^ ‘With twelve people in the waiting room’, elaborated another doctor, ‘no wonder I worry about the half-hour I am giving to the patient who *might* have a *carcinoma of the lung* or the difficult maternity case or anyone who presents a real medical or psychological problem. If I average six minutes per patient I’m very lucky and a busy surgery then lasts two and a half hours’.^62^

Such feelings manifested materially. When a GP felt a patient did not warrant their attention, frustration could lead to lax attention or treatment. This might come in the form of deferring a patient for another time, prolonging the unease prompting attendance. Of nineteen patients that one GP recorded seeing in a surgery session during the mid-1950s, a fifth were—in his words—‘fobbed’ or ‘put off’. Other patients received minimal attention—physical examinations or repeat prescriptions with no further probing, or reassurance offered ‘very scantily’.^63^ The doctor admitted to feeling ‘very sullen about his work that day’, and ‘resented having to be inside on such a lovely afternoon’.^64^ Although making no direct reference to the rush of the open surgery, such recollections highlighted how GPs’ feelings about their work shaped the ‘care’ that might be offered.

This apathy, irritation and stress was, however, historically structured. Frustrations with ‘trivia’ and crowded waiting rooms were often entangled with antipathy towards the NHS and its perceived effects on the work and status of general practice. Although some doctors who had performed significant amounts of NHI work saw little increase in their workload after 1948, this was not universal.^65^ Doctors in suburban and town practice in particular had generally performed greater amounts of private practice, the loss of which made the transition to the NHS much more disruptive.^66^ These practitioners, who tended to be white, male and middle- or upper-middle class, were now exposed to greater demand from patients from a wider range of classes, genders, and ages, while the elimination of financial barriers to seeking care for ‘minor’ conditions was felt keenly.^67^ ‘I have seen in one surgery’, noted one GP in 1949, ‘three common colds, two cases of pimples on the face and one on the abdomen. Not one of these patients would have visited the doctor if the service had not been free’. ‘As it is’, they suggested, ‘patients do not hesitate to wait a long time in the waiting room in order to obtain aspirins’. Together with increased demands for certification, ‘the swamping of surgeries by people with the most trivial conditions’ was ‘stultifying medicine so that one has not the time nor the mental alertness to deal with those who are really ill’.^68^

Complaints about ‘free treatment’ continued into the 1960s. GPs in one study estimated a fifth of their surgery consultations were for ‘trivial, unnecessary or inappropriate reasons’.^69^ Although this investigation found that 52 per cent of respondents enjoyed general practice ‘very much’, and 37 per cent enjoyed it ‘moderately’, a vocal minority were deeply critical about their status.^70^ Concerns about triviality were, again, connected to anxieties around the status and identity of practitioners and patients. Alongside criticism of undeserving ‘neurotics’, complainants framed their dissatisfaction through the imagery of dirt and unruliness central to racialized framings of class and racist discourses of immigration.^71^ One respondent suggested that the GP ‘was more of a waste product of the medical schools than an end product’, whilst another linked being ‘swamped with trivialities’ with the ‘utter futility and humiliation of a professional man who feels his training is wasted’.^72^ In other words, feelings of irritation, humiliation and stress ‘stuck’ to the figure of the ‘trivial’ patient because of what they said to a certain section of a traditionally white, male and middle-class profession.^73^

Of course, like much of the post-war British public, doctors were accustomed to surveys, and as their complaints required ‘going public’ they possessed a strong performative edge.^74^ Nonetheless, this did not necessarily undermine their earnestness. As ‘public articulation[s] of private grievances’, Daisy Payling has argued, complaints provided windows onto often-shared, deeply felt ‘“anxieties, doubts and frustrations”’.^75^ Similarly, as public objection generally followed unmet expectations, it also articulated values integral to complainants’ sense of identity. For aggrieved GPs, frustrations with the NHS, their status, and the trivia of ‘open’ surgeries were simultaneously felt acutely and politically mobilized.^76^ Doctors wielded such criticisms in campaigns over working conditions, but these complaints also fuelled conservative GPs’ organized opposition to the NHS and emigrations overseas.^77^ As will now be seen, doctors’ affective responses to these entangled issues also motivated them to deploy appointment systems to regulate the time of surgery work.

## Appointment Systems

For many GPs, social commentators and patient representatives, appointment systems provided a necessary corrective to the faults of open surgeries, at least initially. Systems varied but shared the basic mechanics. To consult a GP, patients were asked to no longer simply attend during surgery hours, but to make an appointment first (by telephone or in person). Once at the surgery, patients would wait by book rather than order of arrival. And if the doctor wanted another consultation, receptionists organized this as patients left.^78^

Change was gradual at first as appointment systems needed new resources. Particularly in larger practices, extra telephone lines were often necessary.^79^ Administrative materials were also required—at minimum, books to record and organize the day and week’s work, but some practices also used portable cards to remind patients of both the rules of the system and their next appointment.^80^ Crucially, as discussed below, they required clerical staff—receptionists handled calls, booked patients (according to unstated norms), prepared records, and managed disgruntled patients.^81^ Finally, spatial reorganization within practices was necessary to allow for desks, offices and new storage spaces.^82^ GPs grumbled about the expense of such changes.^83^ Until doctors and the Ministry of Health agreed a new contract in 1966, GPs paid directly for new staff and materials, expected expenses having been averaged out in capitation fees.^84^ As such, by the early 1960s only around 6 per cent of practices employed full-time systems.^85^ Nonetheless, after 1966—when the Ministry reimbursed a substantial proportion of costs—take-up accelerated considerably nationwide, and between two thirds and four-fifths of practices operated systems by 1974.^86^

Although GPs regularly suggested that full-time appointments systems improved their affective experience of surgery work, this was not always the primary rationale. Whether performative or sincere, advocates generally referred to the advantages to patients of reduced waiting, fixed times of consultation and the reduced risk of infection in less crowded spaces.^87^ In this sense, despite a developing psychological literature on the subjective experience of waiting, social, economic and medical rationales provided the explicit foundation for reform.^88^ Similarly, improved efficiency and planning remained central for GPs, too. For a minority of practitioners, appointment systems explicitly formed part of a broader interest in efficient organization that borrowed analytic frameworks from industry.^89^ Most, however, simply praised their new ability to know what work lay ahead, manage weekly workflow, and reduce time-costly visiting or overall consultation rates.^90^ Crucially, this meant that temporal inputs might be reduced, or kept consistent but deployed to greater effect.^91^

Questions of productivity were not, however, divorced from questions of affective life. Advocates for appointments suggested the more ordered and efficient use of surgery time reduced physically and mentally taxing conditions inside the practice. As noted above, many GPs experienced more managed workflows as an ‘immediate lightening of the strain and burden of work’.^92^ For some, this improvement related to how new systems removed the ‘forbidding sight’ of the full waiting room.^93^ With appointments, one GP observed, ‘one tends to lose the feeling of pressure from a waiting room full of a vast unknown population’.^94^ For others, control over working hours—even being able to finish ‘very near the scheduled time’—relieved frustrations with ‘interminable’ open surgeries.^95^ Equally, it created time that might be reserved for social occasions, professional development or leisure pursuits. Rather than ‘seeing patients for at least an hour after the nominal closing time’, surgeries could now end as scheduled. ‘Special occasions such as meetings and holidays’ could be ‘arranged in advance’.^96^ Similarly, ‘if the doctor wishes to go out for dinner he can arrange for his appointments to be early; on the other hand, he can arrange to start late if he knows he cannot be back for evening surgery on time’.^97^ Especially when co-ordinated with a rota system, then, appointments allowed for ‘time off’.

Facilitating ‘work-life’ distinctions was not trivial. Particularly in industrial settings, general practice was physically and emotionally punishing. One medical union argued to a 1958 Royal Commission that 24/7 responsibility for patients made a doctor’s life ‘one of constant anxiety’.^98^ Satirical observations that general practitioners all ‘drop dead at forty through overwork’ may have been written for comic effect.^99^ However, they rang true enough, and a substantial minority turned to dangerous coping mechanisms.^100^ One account of practice in the Welsh valleys noted how ‘one doctor was drinking hard and had an ulcer, another died of a heart attack …. Another one worked too hard in an epidemic and [took their own life] a fortnight later’.^101^ Appointment systems were clearly an inadequate response to such challenges. Together with other technologies of temporal regulation such as rotas and deputizing services, though, they could simultaneously facilitate lifestyle choices and provide relief from the strains of service for doctors and their families.^102^

Finally, advocates’ regular emphases on control over surgery hours implicitly tied questions of temporal autonomy to concerns about professional and social status. GPs disgruntled by the NHS’s universality regularly bemoaned what they saw as patients’ newly entitled attitudes. They grumbled about a minority treating them as a ‘servant’ or ‘supplier of medicine’, whilst criticisms of patients who asserted their rights—who ‘tell us what is wrong and what he wants for it’—were widely repeated.^103^ Again, questions of time were prominent. Demanding patients ‘abused’ the service, bringing trivial complaints at inconvenient and self-indulgent times—an ‘ordinary cold’ became ‘an occasion for a visit, a feverish cold for a night call’.^104^ Doctors whose self-image had been shaped by class privilege and professional socialization thus strongly resented what they saw as a one-sided relationship. As one GP bewailed in the 1960s: ‘people have an increasing belief that they have a right to a doctor’s services for anything at any time of the day or night and can have him over a barrel if he doesn’t do what they want. But if they abuse the service there is no redress except to get rid of the patient and that’s slitting your own throat.’^105^ By enabling surgeries to operate ‘to a time of the *doctor’s* own making’, appointment systems allowed GPs to reclaim some of this (imagined) lost status relative to their increasingly undeferential patients.^106^

Perhaps ironically, patients initially strongly supported new arrangements; emergent consumer groups even campaigned for them.^107^ Although mediated, surveys undertaken by doctors, sociologists, and patient-consumer organizations in the 1960s reported approval ratings between 70 and 95 per cent.^108^ Similarly, early assessments of appointment systems found a high percentage of patients attending by appointments, demonstrating support through action.^109^ Where patients explained their positive attachments, reduced surgery waiting and the capacity to plan other tasks around the fixed appointment were prominent. ‘Against the trouble of making an appointment’, wrote the Chair of the Patients’ Association to the *Guardian* (1963), ‘must be set the saving of perhaps a hour or more in the waiting room’.^110^ Sociological investigation noted some class differences in support for, and use of, appointments. Generally these differences mapped onto structural factors, such as access to telephones and transport, but around 70 per cent of working-class respondents reported as satisfied with their appointment systems.^111^ Nonetheless, the perceived benefits for some patients reflected their social positionality. As one patient wrote to the *Guardian* in praise of a GP’s ticketing system, the arrangements facilitated the lifestyle and responsibilities of the non-waged, presumably white British middle-class housewife.^112^ Patients, according to this correspondent, avoided a ‘waste of [their] time’ and could ‘go shopping, have a coffee, return home and do a little housework etc’.^113^

Such framings demonstrate how irritations with delay—and the relief and appreciation felt following its reduction—assumed particular hues in the post-war decades. Undoubtedly, the reference to time being ‘wasted’ and ‘saved’ aligns with contemporary observations on frustrations with delay under modern capitalism—where timetabled productivity and accelerated social life pull the subject in multiple directions and time is economized as a resource to be spent.^114^ Simultaneously, however, gendered experiences of temporal conflict were articulated in reference to women’s expected reproductive labour, a cultural norm tied to heteronormative projects of family stability in post-war reconstruction, employment and welfare.^115^ Indeed, such tensions were particularly acute for mothers of young children who waited with, and for, their children more often than fathers. They grew critical of GPs who did not provide materials to occupy their notoriously disruptive and creative waiters.^116^ Of course, appointments could create their own problems for working mothers, who might need to attend during lunch hours or between shifts.^117^ But surveys nonetheless found mothers of young children among the strongest supporters of appointments, praising the way that appointments meant they ‘hadn’t to control children for long periods’.^118^

Patients’ acceptance of appointments might also have reflected the shifting cultural associations of queuing with national decline and political failure, as well as patients’ own growing familiarity with technologized and managed waiting across different areas of economic and social life. As Joe Moran has noted, between the 1960s and 1980s waiting itself was transformed through political, managerial and technological ‘revolutions’.^119^ Waiting in banks, Post Offices and train stations, for instance, was gradually reorganized by new queuing systems, and particularly by automation—in the form of ATMs, ticket machines, and stamp dispensers. Likewise, supermarkets aimed to shorten queues at checkouts and elsewhere through ticket dispensers, conveyer belt technologies, and greater staff investment.^120^ As Moran has noted, such transformations were uneven. The bus stop, for instance, used most by elderly, female, working-class and migrant passengers, experienced little ‘revolution’.^121^ Nonetheless, changes were far reaching. Even the historic ‘dole queue’ was gradually given new meanings, as giro cheques replaced cash benefits, and physical queues at Labour Exchanges were replaced by managed and seated waiting areas at Job Centres.^122^

As GPs noted, appointments formed part of these transformations in waiting, becoming increasingly common in fields as diverse dentistry and hairdressing after the 1950s.^123^ But they did not always generate the affects that doctors desired. GPs maintained that the increased scarcity and difficulty of securing appointments would enhance patients’ appreciation of the doctor’s time. Some felt this would deter trivial patients, others that consulting patients would give greater weight to advice offered in this ‘special time’.^124^ Scarcity, though, had unintended consequences. Especially into the 1970s, appointment systems seemingly became less effective, creating barriers to consultation. Sociological research suggested that only 63 per cent of patients were able to get an appointment within 24 hours, and that around 12 per cent of patients whose GPs ran appointments systems deferred visiting their doctor due to the need for an appointment.^125^ Notably, postponement was significantly more common among patients who struggled to get appointments within 2 days of calling.^126^ ‘Difficulties in providing enough time in the consultation’, suggested *The Lancet*, ‘seem to have been exchanged for difficulties in seeing the doctor soon enough’.^127^ Under some conditions, delay, rather than acceleration, became the appointment’s dominant temporality.

For patients unable to be seen quickly, appointments extended, rather than resolved, anxious anticipation and waiting for care. Not only was the surgery wait itself a period of trepidation, but patients had often waited in uncertainty before deciding to consult a GP—whether to see if symptoms passed, or from concern for ‘troubling’ the doctor.^128^ As one GP proposed, patients could ‘contain symptoms for a certain length of time’ (often days, sometimes months) but ‘at a certain point they can no longer do so’.^129^ For patients who then struggled to get an appointment swiftly, a small minority resorted to attending Casualty and other hospital departments.^130^ For many others, anxieties—alongside anger at broken promises of accessibility—could manifest as criticisms of new systems. Critics considered the ‘previous system’ of ‘waiting one’s turn’ when ill to be ‘much more humane’ as it enabled earlier access to treatment.^131^ One patient foregrounded the distress of deferral in a submission to the Patients’ Association: they ‘would prefer to wait in the surgery, however long, rather than spend three days building up nervous tension’.^132^ Lamentations, however, were rarely so depersonalized. A critical minority often targeted receptionists for mediating access to doctors’ time and their right to timely medical attention.

## The Old Times and the New: Receptionists and the ‘Family Doctor’

Into the 1970s, national surveys of patient satisfaction with general practice returned overwhelmingly positive headline figures.^133^ Even as satisfaction with appointment systems dipped, over two-thirds of patients nationally preferred appointments, and more locally when delays were short.^134^ And yet, impressionistic journalism and small-scale consumer research returned consistent complaints about GPs’ accessibility, and particularly difficulties with receptionists. Not all complainants wanted a reversion to older forms of organization. Patient groups generally wanted improvements.^135^ But criticisms clearly expressed a sense of loss for what ‘modern’ general practice allegedly left behind.

Dissatisfaction with appointment systems formed only part of wider concerns with ‘modern’ healthcare and society. Over the 1960s and 1970s, associations were forged between appointment systems, forms of collective practice, and ‘new’ or ‘progressive’ care centred on improved organization.^136^ Predicated on the calculable, ordered and standardized time of the clock, appointment systems easily assumed qualities of modernity.^137^ But connections with modernization went further. Although the 1966 GP contract was provoked, in part, by long-term professional dissatisfaction with working conditions and status, it was agreed in the context of expanding state finance for modernizing healthcare infrastructure and the British economy.^138^ The Hospital Plan, designed to transform the nation’s outdated hospital stock, was launched 4 years earlier.^139^ And new state investment significantly boosted the creation of appointment systems, as well as primary care teams, purpose-built surgeries and other organizational technologies.^140^

Taken together, ‘modernisation’ amounted to, for some, reduced personal contact with their doctor. ‘Nobody disputes’, noted one reporter in 1973, ‘that doctors are less personally available than they used to be’.^141^ Anxieties about such developments were not new. Bemoaning financial and political support for group practice and health centres in 1965, a correspondent for *The Times* railed against the day when ‘professional politicians, aided by doctrinaire doctors, will have squeezed out of existence the single-handed practitioner’. That would be ‘a pity’ because ‘they represent a long tradition of committed, conscientious personal relationship and service which nothing will replace’.^142^ Some GPs were equally uneasy. They worried that appointment systems could create ‘barriers’ to patients, preventing early consultation and diagnosis of serious illness.^143^ Others maintained that skill and ‘a sound understanding of the doctor-patient relationship’ was more important to good general practice than facilities and organization more akin to ‘hospital medicine’.^144^

But for some critics, concerns about change tapped into broader anxieties, especially around the loss of imagined social and cultural idylls at the heart of visions of (white) Britishness.^145^ For instance, one columnist lamented that the retirement of their GP represented the breakdown of ‘traditional’ social structures and growing anonymity of modern life.^146^ Asking how patients ‘can extend the same frankness to a group practice as to an individual doctor’, the author regretted that ‘with the passing of the family doctor, the relationship between family and doctor is being lost’. This passing was merely one among many: the once-familiar figure of the local the vicar had faded from social prominence, and once-esteemed schoolteachers now resorted to radical strike action. ‘Old values change’, they surmised, ‘personal contact decreases. Respect diminishes into disrespect, which is not always healthy’.^147^

As a ‘symptom’ of modern life, then, concerns with appointment systems fed wider concerns, particularly those about the fate of the ‘family doctor’. Although the ‘family doctor’, as an omnipotent, tirelessly devoted, ever-available family friend and teacher, was likely more imagined than lived, this figure retained considerable cultural purchase in the post-war period.^148^ Family doctors provided subjects of health magazines, and popular fiction and television shows, whilst the closely-connected ‘country doctor’ received photographic and poetic peons to their work.^149^ Crucially, as Chelsea Saxby has argued, this nostalgic mourning for ‘a socially familiar and personally concerned family doctor’ provided a profound and ‘productive vehicle for patients to express their concerns about healthcare’ in the present: most prominently, a perceived loss of direct access to a known GP.^150^

It is within this context that the intermediary figure of the receptionist as the object of patients’ aggression and disdain makes sense. Their visibility and centrality to appointment systems made receptionists the system’s human face. Doctors were aware that receptionists could generate resentment as a result, and that they would have to manage waiting patients’ emotions as much as the appointment book. GPs thus gave the character and temperament of the receptionist deep consideration. ‘Patients’, noted one reflection, ‘are likely to react strongly against an unfriendly or harassed bureaucracy’.^151^ Receptionists needed to be ‘calm, kind, efficient, and, occasionally, firm’ where needed.^152^ According to one handbook, they should ‘like people’, ‘have a pleasant manner’ and ‘be understanding’. Alongside ‘reticence’, ‘tact’ and ‘a sense of humour’, these sensibilities would enable receptionists to deal ‘pleasantly with all types of patient on the telephone—the querulous patient, the anxious patient, even the aggressive patient’.^153^ Patients were, after all, already experiencing nervous uncertainty about their health, and receptionists were to be reassuring ‘angels in the house’.^154^

Indeed, although psychosocial perspectives were slowly reshaping how some GPs understood their work into the 1960s, this emphasis on emotional labour meant doctors consistently saw reception work as a job for women.^155^ These same sexist attitudes constructed the types of emotions—especially resentment, anger, fear, even hate—that ‘stuck’ to receptionists. Such emotional patterning was clearest in the press. Misogynistic language—of ‘too bossy girls’—provided the basis for headlines, and columnists pilloried reception staff as ‘dragon-like’ or compared them to ‘mean-tempered terriers’.^156^ Together with criticism of the supposed ‘obstruction by lay staff with a power complex’, such frameworks underlined how negative affective associations were formed from the intersectionality of receptionists’ gender and their lower middle-class roles as bureaucrats.^157^

Despite most patients believing receptionists played an important part in ‘the smooth working of the practice’, these cultural tropes influenced the grievances that patients felt and reported to consumer bodies, researchers and GPs.^158^ Into the 1970s, many patients considered medical troubles to be private, something ‘between your doctor and yourself’. When receptionists asked about the reason for seeing the doctor, patients thus resented the officious ‘intrusion’.^159^ To people in pain, or anxious about their physical or mental state, the appointment was a promise of attention, as well as of care that was now their social right.^160^ ‘Barriers’ were, therefore, most unwelcome—especially when presented in the form of staff who deviated from expected submissiveness, or who supposedly lacked the ‘training’ and status expected in decisions of medical triage.^161^ Similarly, where explaining problems might be felt by patients as ‘intimidating’ or ‘embarrassing’, criticisms focused on receptionists’ comportment—their lack of ‘courtesy and consideration’.^162^ Again, these grievances were filtered through gendered and classed tropes of ‘gossipy’ and ‘snobbish’ middle-class women: ‘obstructive’ and ‘indiscreet’ receptionists who ‘spoke loudly about their [patients’] problems in front of a waiting-room full of people’ were particularly criticized.^163^

As with the development of appointment systems more broadly, these experiences, discursive creations, and affective relations all influenced the everyday functioning of the health service, as well as how patients and staff felt about and experienced their work and care. As discussed, some patients delayed contacting the doctor due to difficulties getting an appointment and negotiating with reception staff. Equally, just as patients’ appreciation of the temporal autonomy and reduced waiting afforded by appointments had ensured most complied with new systems, a rejection of the new order could be fuelled by frustration or disappointment with its regulations and policing. Throughout the early operation of systems, patients continued calling outside of requested hours to make appointments, sometimes necessitating employment of more staff.^164^ Similarly, published reports referred to a core of patients who refused to make appointments and simply attended the surgery.^165^ Most systems endured, but a minority of practitioners blamed ‘non-cooperation, unpunctuality, or queue-jumping’ for the failure of new arrangements.^166^ One practitioner suggested that their dysfunctional system created more ‘strain’ than open surgery hours.^167^ Given that around 15 per cent of systems in the mid-1960s were abandoned, patient non-compliance made a not inconsiderable impact.^168^

## Conclusion

Although pervaded with unintended consequences, the emergence of appointment systems over the first three decades of the NHS substantially transformed how time was ordered—and how patients waited—within post-war general practice. Open surgery sessions had been, in some sense, emblematic of the earliest temporal promises of the newly expanded health and welfare services; doctors, as state-contracted professionals, were made accessible whenever patients were in need, regardless of financial circumstances. A manifestation of ‘cradle to grave’ availability in the everyday.^169^ Inequalities of access existed in practice. Rural remoteness, or class, age, and gender inequities in mobility, for instance, might structure the capacity to attend surgery and wait.^170^ Nonetheless, this openness marked temporal rights indicative of a hoped-for universalism of welfare.

Appointment systems, however, were developed to address the perceived deficiencies of open surgeries for doctors and patients. For GPs, open surgeries were often experienced as an unmanageable torrent, creating anxieties, guilt and feelings of stress, strain and exhaustion. They intended appointments to regulate the flow of work, enhance doctors’ autonomy and working conditions, and even create new forms of leisure and family time. In a profession still dominated by white, male and middle-class practitioners, moreover, some even fantasized that new systems would deter the trivial, and recurrent attender who offended doctors’ (often fragile) self-image. Patients—particularly women and mothers—also harboured hopes that appointments would enhance a control of time already compressed by competing demands of waged work and unwaged reproductive labour.^171^ But, perhaps as importantly, patients were attracted to the promise of reduced waiting in the surgery, a wait that could be experienced as interminable, boring, frustrating and burdensome, as well as pleasurable or caring.

Financial support from a ‘modernising’ state sparked the widespread up-take of appointment systems, which had been supported, even promoted, by patients increasingly accustomed to managed, technologized forms of waiting. For many patients and GPs, new systems met their promises, introducing a reassuring sense of temporal autonomy and reducing the surgery wait. Class and age-related inequalities persisted into the 1980s, but systems retained broad support and proved flexible enough to function. As appointment intervals were gradually extended, some GPs even altered how they spent time with patients.^172^

Change, of course, was uneven. By the 1980s, a small number of GPs—mostly in rural and small-town settings lacking hospital facilities—retained older forms of organization.^173^ Moreover, even where appointments and other forms of temporal and organizational modernization were established, GPs’ irritations with ‘trivial’ or recurrent patients remained. And for patients, whether attending a ‘modern’ GP or not, satisfaction with their general practice service remained high.

Nonetheless, as an intensifying cultural nostalgia for the known and ever-available ‘family doctor’ suggested, appointments also remade the very affective and material circumstances from which they emerged. They introduced new frustrations, anxieties, and stresses into attending the doctors’ for some, and—in remaking expectations and experiences of provision—simultaneously altered how patients and doctors related to one another. Crucially, patients were no longer guaranteed immediate attendance at surgery hours, but could now be deferred for a ‘reasonable’ period, as defined by the doctor.^174^ Although not explicitly articulated by patients within a discourse of citizenship, these shifting temporal rights were keenly felt by those forced to wait outside the surgery for days or weeks, unable to consult the doctor when needed. Structured by classed and gendered norms, the now-prominent figure of the receptionist also became the focus for consternation at delay, and frustration at bureaucratic obstacles being erected between patient and practitioner.

Such changes altered not only the affective landscape of the surgery, but also the interactions that took place within and without. Some patients simply deferred attending surgery due to the logistical and emotional challenges of engaging with new systems. Others, perhaps unwittingly, revived older practices of directly attending hospital, out of anxious uncertainty and frustration with the delays that appointments imposed.^175^ Others still continued to attend without appointments, placing a minority of GPs under greater strain than open surgeries, and forcing further innovations in temporal regulation.^176^ Public and official complaints about new systems buttressed non-cooperation and, ironically, as some systems broke down, waiting in the surgery could take on the lengthy waits of open surgeries.^177^ Only now, as one GP noted in 1992, frustrations oriented towards unmet promises: ‘I don’t mind waiting an hour. But I object to waiting two hours when I have had an appointment’ for a specified time.^178^

The transformation of time in general practice therefore had extensive everyday repercussions. As yearnings for traditional ‘family doctors’ and the ‘comraderie’ of the open surgery suggested, vocal—often socially conservative commentators and patients—began to relate to general practice as ever-more impersonal and remote.^179^ As general practice became ‘modernised’, that is, it implicitly lost what distinguished it from other public services (or even hospitals) for these critics. And as the most frequently-contacted site of healthcare in the post-war period, by definition this had repercussions for everyday experiences of the NHS overall. This was not necessarily universal. Doctors into the 1990s still recalled having incredibly close and rewarding relationships with their pati ents.^180^ But for even the most satisfied doctors and patients, the surgery session itself assumed different meanings, expectations and practices over the first three post-war decades.

## Footnotes

1 Mass Observation, *Meet Yourself and the Doctor’s* (London, 1949), 41–46.

2 Anne Digby, *The Evolution of British General Practice, 1850-1948* (Oxford, 1999), 140.

3 Stephen J. Hadfield, ‘A Field Survey of General Practice, 1951-2’, *British Medical Journal* [*BMJ*], 26 September 1953, 701; ‘14 Days to See Your Doctor’, *The Lancet*, 28 April 1973, 923.

4 My use of ‘everyday’ aligns with historical explorations of social practices—their performance and meanings—that had been rendered culturally routine and mundane, as opposed to exceptional events, institutions or ‘high politics’. On theorizations of the everyday and its affective life: Ben Highmore, *Everyday Life and Cultural Theory: An Introduction* (London, 2002); Ben Highmore, *Ordinary Lives: Studies in the Everyday* (London, 2010).

5 Laura Salisbury, ‘“Between-time stories”: Waiting, War and the Temporalities of Care’, *Medical Humanities*, 46 (2020), 96-06, 99. Though note minor exceptions: Ann Cartwright and Robert Anderson, *General Practice Revisited: A Second Study of Patients and Their Doctors* (London, 1981), 29–30.

6 John McCraith, ‘Appointment Systems in General Practice’, *BMJ*, 30 January 1965, 320.

7 British Library, London, Oral History of General Practice, C648/06, ‘Margaret Norton interviewed by Michael Bevan’, 1992, Part 4 c. 0m 45s.

8 David Armstrong, ‘Space and Time in British General Practice’, *Social Science and Medicine*, 20 (1985), 659–66. On ‘temporal architectures’: Sarah Sharma, *In the Meantime: Temporality and Cultural Politics* (Durham, 2014).

9 Barbara Adam, *Timewatch: The Social Analysis of Time* (Cambridge, 1995), 59–66, 73–76, 85–94.

10 Steve Sturdy and Roger Cooter, ‘Science, Scientific Management and the Transformation of Medicine in Britain, c.1870-1950’, *History of Science*, 36 (1998), 421–66.

11 Stephen Kern, *The Culture of Time and Space, 1880-1918* (London, 1983).

12 E. P. Thompson, ‘Time, Work-Discipline, and Industrial Capitalism’, *Past and Present*, 38 (1967), 56–97; Eviatar Zerubavel, *Hidden Rhythms: Schedules and Calendars in Social Life* (Chicago, 1981).

13 Salisbury, ‘Between-time Stories’, 98.

14 On entanglements of technologies and time: Judy Wajcman, *Pressed for Time: Acceleration of Life in Digital Capitalism* (Chicago, 2015).

15 Florence Sutcliffe-Braithwaite, *Class, Politics, and the Decline of Deference in England, 1968-2000* (Oxford, 2018); Angela Davis, *Modern Motherhood: Women and Family in England, 1945-2000* (Manchester, 2012); Helen McCarthy, *Double Lives: A History of Working Motherhood in Modern Britain* (London, 2020); Paul Gilroy, *Ain’t No Black in the Union Jack: The Cultural Politics of Race and Nation* (London, 1987); Jon Lawrence, *Me, Me, Me? The Search for Community in Post-War England* (Oxford, 2019); Frank Mort, Becky Conekin, and Chris Waters, eds, *Moments of Modernity: Reconstructing Britain, 1945-64* (London, 1999).

16 Ghassan Hage, ‘Introduction’, in Ghassan Hage, ed., *Waiting* (Melbourne, 2009), 5.

17 Laura Tanner, *Lost Bodies: Inhabiting the Borders of Life and Death* (Ithaca, 2006), 64–80; Sally Sheard, ‘Space, Place and (Waiting) Time: Reflections on Health Policy and Politics’, *Health Economics, Policy, and Law*, 13 (2018), 226–50; Caitjan Gainty, ‘Why Wait?’, *Modern American History*, 2 (2019), 249–55.

18 On such diverse meanings in contemporary life: Lisa Baraitser, *Enduring Time* (London, 2017).

19 Joe Moran, ‘Queuing Up in Post-War Britain’, *Twentieth Century British History*, 16 (2005), 283–305.

20 Agnes Arnold-Forster, ‘“A small cemetery”: Death and Dying in the Contemporary British Operating Theatre’, *Medical Humanities*, 46 (2020), 278–87; Jack Saunders, ‘Emotions, Practices and the Changing Composition of Class, Race and Gender in the National Health Service, 1970-79: “Lively discussion ensued”’, *History Workshop Journal*, 88 (2019), 204–28; Yasmin Gunaratnam, *Death and the Migrant: Bodies, Borders and Care* (London, 2013); Stephen Brooke, ‘Space, Emotions and the Everyday: The Affective Ecology of 1980s London’, *Twentieth Century British History*, 28 (2017), 110–42; Julian Simpson, *Migrant Architects of the NHS: South Asian Doctors and the Reinvention of British General Practice* (Manchester, 2018).

21 Elizabeth Toon, ‘“Cancer as the general population knows it”: Knowledge, Fear, and Lay Education in 1950s Britain’, *Bulletin of the History of Medicine*, 81 (2007), 116–38; Andrew Seaton, ‘Against the “sacred cow”: NHS Opposition and the Fellowship for Freedom in Medicine, 1948–72’, *Twentieth Century British History*, 26 (2015), 424–49; Jennifer Crane, ‘“Save our NHS”: Activism, Information-based Expertise and the “new times” of the 1980s’, *Contemporary British History*, 33 (2019), 52–74; Hannah J. Elizabeth, Gareth Millward and Alex Mold, ‘“Injections-while-you-dance”: Press Advertisement and Poster Promotion of the Polio Vaccination to British Publics, 1956-1962’, *Cultural and Social History*, 16 (2019), 315–36.

22 As in: Brooke, ‘Space, Emotions and the Everyday’; Joanna Bourke, ‘Fear and Anxiety: Writing about Emotion in Modern History’, *History Workshop Journal*, 55 (2003), 123.

23 Ruth Leys, ‘The Turn to Affect: A Critique’, *Critical Inquiry*, 37 (2011), 434–72.

24 Brooke, ‘Space, Emotions and the Everyday’; Elizabeth et al., ‘“Injections-while-you-dance”’. Elizabeth’s theoretically rich work pushes this approach into popular culture, care and public Health governance: ‘*Love Carefully* and without ‘over-bearing fears’: the Persuasive Power of Authenticity in late 1980s British AIDS Education Material for Adolescents’, *Social History of Medicine*, (2021), 1–26.

25 Jan Plamper, ‘The History of Emotions: An Interview with William Reddy, Barbara Rosenwein and Peter Stearns’, *History and Theory*, 49 (2010), 237–65; Thomas Dixon, *From Passions to Emotions: The Creation of a Secular Psychological Category* (Cambridge, 2006); Rhodri Hayward, ‘The Pursuit of Serenity: Psychological Knowledge and the Making of the British Welfare state’, in Sally Alexander and Barbara Taylor, eds, *History and Psyche: Culture, Psychoanalysis and the Past* (Basingstoke, 2012), 283–304.

26 Sara Ahmed, *The Cultural Politics of Emotion* (Edinburgh, 2014).

27 Brooke, ‘Space, Emotions and the Everyday’.

28 Ahmed, *The Cultural Politics of Emotion*.

29 On emotion’s centrality to understanding subject-object attachments: Ahmed, *The Cultural Politics of Emotion*, 12.

30 Digby, *The Evolution of British General Practice*, 231.

31 Digby, *The Evolution of British General Practice*, 321.

32 Martin Gorsky, ‘Friendly Society Health Insurance in Nineteenth-Century England’, in Martin Gorsky and Sally Sheard, eds, *Financing Medicine: The British Experience Since 1750* (London, 2006), 147–64.

33 Anne Digby, ‘The Economic and Medical Significance of the British National Health Insurance Act, 1911’, in Martin Gorsky and Sally Sheard, eds, *Financing Medicine: The British Experience Since 1750* (London, 2006), 182–98.

34 Digby, *The Evolution of British General Practice*, 149.

35 Digby, *The Evolution of British General Practice*, 321. On GPs and NHI: Digby, ‘The Economic and Medical Significance of the British National Health Insurance Act’, 186–90.

36 Peter Black, ‘They Also Suffer Who Only Sit and Wait’, *Daily Mail*, 5 November 1955, 4.

37 John Crosby, ‘The Medicine Man’, *The Guardian*, 29 December 1974, 6.

38
*One Night I Danced with Mr Dalton*, ABC Television (1968); Buchi Emecheta, *Second-Class Citizen* (London, [1974] 1987), 112.

39 Respectively: John Cunningham, ‘Doctors Avoid ‘unnecessary’ Calls’, *The Guardian*, 2 May 1973, 7; Retired GP, ‘Waiting for the Doctor’, 26 October 1954, 6.

40 Stephen Taylor, *Good General Practice: A Report of a Survey* (London, 1954), 50–1; Nick Bosanquet and Chris Salisbury, ‘The Practice’, in Irvine Loudon, John Horder and Charles Webster, eds, *General Practice under the National Health Service, 1948-1997* (Oxford, 1998), 53.

41
*Meet Yourself and the Doctor’s*; A Patient, ‘A Morning in a Doctor’s Surgery’, *The Practitioner*, 178 (1957), 355–6.

42 Harold Schweizer, *On Waiting* (London, 2008); Claire Langhamer, ‘Mass Observing the Atom Bomb: The Emotional Politics of August 1945’, *Contemporary British History*, 33 (2019), 208–25. Existentialist interpretations of *Waiting for Godot* dominated this period: Laura Salisbury and Lisa Baraitser, ‘Depressing Time: Waiting, Melancholia, and the Psychoanalytic Practice of Care’, in Elisabeth Kirtsoglou and Bob Simpson, eds, *The Time of Anthropology: Studies of Contemporary Chronopolitics* (London, 2020), 103.

43 ‘Why Are We Waiting?’, *Platform* (Basingstoke, 1964), 21.

44
*Meet Yourself and the Doctor’s*, 46.

45 Gerry Stimson and Barbara Webb, *Going to See the Doctor: The Consultation Process in General Practice* (London, 1975), 25–26.

46 Diana Rowntree, ‘Therapy in the Waiting Room’, *The Guardian*, 4 November 1959, 6; Martin D. Moore, ‘“Bright-while-you-wait”? Waiting Rooms and the National Health Service, c. 1948-1958’, Jennifer Crane and Jane Hand, eds, *Posters, Protests and Prescriptions: Cultural Histories of the National Health Service in Britain* (Manchester, Forthcoming).

47 Salisbury, ‘Between-time stories.

48 Mass Observation, ‘Queuing’, September 1948. Report 3036. <http://www.massobservation.amdigital.co.uk.uoelibrary.idm.oclc.org/Documents/Details/FileReport-3036>.

49 Mass Observation, 1, 5–6; A Patient, ‘A Morning in a Doctor’s Surgery’, 356.

50 See newspaper clipping on file: The National Archives, London, MH 135/255, Lanarkshire Patient, ‘Visiting the Doctor’, *Glasgow Herald*, 30 June 1955; Digby, *The Evolution of British General Practice*, 140–2.

51 And as one GP noted, most patients were willing to wait in an emergency because they knew it could be them one day: BL, Oral History of General Practice, C648/47, ‘Clifford Aston interviewed by Michael Bevan’, 1992, Part 7 c. 24m.

52 Moran, ‘Queuing up in Post-War Britain’.

53 Mass Observation, ‘Queuing’, 4.

54 M. I. Forster, ‘On the Bus’, *Daily Mail*, 24 August 1955, 4.

55 Moran, ‘Queuing up in Post-War Britain’, 284–94; James Hinton, ‘Militant Housewives: The British Housewives’ League and the Attlee Government’, *History Workshop Journal*, 38 (1994), 129–56.

56 Patient, ‘Doctors’ Waiting-Rooms’, *Manchester Guardian*, 18 October 1954, 4.

57 Taylor, *Good General Practice*, esp. xxii–xxiii; Steffan Blayney, ‘Industrial Fatigue and the Productive Body: The Science of Work in Britain, c. 1900-18’, *Social History of Medicine*, 32 (2019), 310–28.

58 Mark Jackson, *The Age of Stress: Science and the Search for Stability* (Oxford, 2013).

59 John Fry, J. B. Dillane, Lionel Fry, Peggy Blake, and James Fry, ‘Appointments in General Practice: Doctors’ and Patients’ Views’, *The Lancet*, 24 August 1963, 398.

60 ‘“Low standard” of Surgeries and Waiting Rooms’, *Manchester Guardian*, 29 June 1954, 4; J. M. Bevan and G. J. Draper, *Appointment Systems in General Practice* (Oxford, 1968), 42–43.

61 A. E. de la T. Mallett, ‘An Appointment System in General Practice’, *The Practitioner*, 184 (1960), 490.

62 ‘How Sick is the Health Service?’, *Daily Mail*, 30 June 1958, 4. Original emphasis.

63 Michael Balint, *The Doctor, His Patient and the Illness* (London, 1957), 48–50.

64 Balint, *The Doctor, His Patient and the Illness*, 48.

65 Richard M. Titmuss, *Essays on the Welfare State* (Bristol, [1958] 2018 ), 114–15, 136–45.

66 Though cf: Digby, *The Evolution of British General Practice*, 333–5, 337–8.

67 Industrial and inner-city areas, by contrast, continued to rely heavily on migrant GPs to function. On how racist, gendered and classed professional hierarchies manifested geographically: Simpson, *Migrant Architects of the NHS*.

68 National Health Doctor, ‘Abuses of the Health Service’, *The Guardian*, 18 October 1949, 6. Also: Gareth Millward, *Sick Note: A History of the British Welfare State* (Oxford, 2022).

69 Ann Cartwright, *Patients and their Doctors: A Study of General Practice* (London, 1967), 41.

70 Cartwright, *Patients and their Doctors*, 42–43.

71 Satnam Virdee, *Racism, Class and the Racialized Outsider* (Basingstoke, 2014).

72 Cartwright, *Patients and their Doctors*, 41–42, 58 respectively.

73 Ahmed, *The Cultural Politics of Emotion*. On policing this professional profile: Douglas M. Haynes, *Fit to Practice: Empire, Race, Gender, and the Making of British Medicine, 1850-1980* (New York, 2017).

74 John Clarke, ‘Going Public: The Act of Complaining’, in Jonathan Reinarz and Rebecca Wynter, eds, *Complaints, Controversies and Grievances in Medicine: Historical and Social Science Perspectives* (London, 2015), 259–69.

75 Daisy Payling, ‘“The people who write to us are the people who don’t like us”: Class, Gender, and Citizenship in the Survey of Sickness’, *Journal of British Studies*, 59 (2020), 324.

76 On emotion in politics: Martin Francis, ‘Tears, Tantrums, and Bared Teeth: The Emotional Economy of Three Conservative Prime Ministers, 1951-1963’, *Journal of British Studies*, 41 (2002), 354–87; Deborah Cohen, ‘The Geopolitical is Personal: India, Britain, and American Foreign Correspondents in the 1930s and 1940s’, *Twentieth Century British History*, 29:3 (2018), 388–410.

77 Seaton, ‘Against the “sacred cow”; David Wright, Sasha Mullally, and Mary Colleen Cordukes, ‘“Worse than being married”: The Exodus of British Doctors from the National Health Service to Canada, c. 1955-75’, *Journal of the History of Medicine and Allied Sciences*, 65 (2010), 546–75.

78 For instance: N. C. Horne, ‘An Appointment System for Use in General Practice’, *BMJ* (Supplement, 29 November 1952), 209; J. D. Bottomley, ‘New Surgery Premises for General Practice’, *BMJ* (Supplement, 26 December 1959), 205; Harry N. Levitt, ‘An Appointment System in a Single-Handed General Practice’, *The Practitioner*, 185 (1960), 209–14.

79 Medical World, *Appointment Systems in General Practice* (London, 1961), 6.

80 Horne, ‘An Appointment System for Use in General Practice’, 209; A. E. de la T. Mallett, ‘An Appointment System in a Group Practice’, *College of General Practitioners Research Newsletter*, 3 (1955), 114–15.

81 Levitt, ‘An Appointment System in a Single-Handed General Practice’, 213–14; Medical World, *Appointment Systems in General Practice*, 5–6.

82 P. M. Higgins, ‘Organisation in General Practice: Success of a Shift System’, *The Lancet*, 30 March 1957, 680–2.

83 ‘A Review of General Practice, 1951-2’, *BMJ* (Supplement, 11 September 1953), 125.

84 Geoffrey Rivett, *From Cradle to Grave: Fifty Years of the NHS* (London, 1998), 80.

85 ‘14 days to see your doctor’, 923.

86 Bosanquet and Salisbury, ‘The Practice’, 50–51, 55–56. For contrasting estimates: Office of Health Economics, *The Work of Primary Medical Care* (London, 1974), 12; ‘14 days to see your doctor’, 923.

87 Horne, ‘An Appointment System for Use in General Practice’, 210; John T. Baldwin, ‘Appointment System in General Practice’, *BMJ* (Supplement, 9 January 1960), 11; Medical World, *Appointment Systems in General Practice*, 4.

88 For instance: Lawrence S. Wrightsman, ‘The Effects of Waiting with Others on Changes in the Level of Felt Anxiety’, *Journal of Abnormal and Social Psychology*, 61 (1960), 216–22; Walter Mischel and Ervin Staub, ‘Effects of Expectancy on Working and Waiting for Larger Rewards’, *Journal of Personality and Social Psychology*, 2 (1965), 625–33.

89 Taylor, *Good General Practice*; Armstrong, ‘Space and Time in British General Practice’.

90 Horne, ‘An Appointment System for Use in General Practice’, 210; J. Stevenson, ‘Appointment Systems in General Practice: Do Patients Like Them, and How Do They Affect Work Load?’, *BMJ*, 27 August 1966, 515–18; Bevan and Draper, *Appointment Systems in General Practice*, 25–26; Office of Health Economics, *The Work of Primary Medical Care*, 12. Visiting rates declined rapidly into the 1980s: Bosanquet and Salisbury, ‘The practice’, Appendix D4, 310. However, GPs had consistently queried the effect of appointments on visiting: Fry et al., ‘Appointments in General Practice’, 397.

91 Stevenson, ‘Appointment Systems in General Practice’.

92 Higgins, ‘Organisation in General Practice’, 681.

93 Olga Franklin, ‘Better Health by Appointment: For the Doctors Too’, *Daily Mail*, 31 October 1962, 8.

94 ‘Why are we Waiting?’, 28.

95 Fry et al., ‘Appointments in General Practice’, 398; de la T. Mallett, ‘An Appointment System in General Practice’, 490.

96 Fry et al., ‘Appointments in General Practice’, 398.

97 Baldwin, ‘Appointment System in General Practice’, 11.

98 ‘Holidays with Pay for Doctors Suggested’, *The Guardian*, 17 January 1958, 3.

99 Richard Gordon, *Doctor at Large* (London, 1955), 8.

100 Ali Haggett, *A History of Male Psychological Disorders in Britain, 1945-1980* (Basingstoke, 2015), 93–94, 123–34.

101 Ferris, ‘The Angry GPs’, 21.

102 Taylor, *Good General Practice*, 194–6; Central Health Services Council Standing Medical Advisory Committee, *The Field Work of the Family Doctor: Report of the Sub-Committee* (London, 1963), 28.

103 National Health Doctor, ‘Abuses of the Health Service’, 6; Hadfield, ‘A Field Survey of General Practice’, 699; Cartwright, *Patients and their Doctors*, 57–58.

104 ‘Heard at Headquarters’, *BMJ* (Supplement, 27 November 1948), 194.

105 Cartwright, *Patients and their Doctors*, 58.

106 Horne, ‘An Appointment System for Use in General Practice’, 210. Emphasis added. On broader anxieties about declining deference: Sutcliffe-Braithwaite, *Class, Politics, and the Decline of Deference in England.*

107 On patient-consumerism: Alex Mold, *Making the Patient Consumer: Patient Organisations and Health Consumerism in Britain* (Manchester, 2015).

108 Cartwright, *Patients and their Doctors*, 155; Fry et al., ‘Appointments in General Practice’, 398.

109 Baldwin, ‘Appointment System in General Practice’, 10.

110 Helen S. U. Hodgson, ‘GPs and Patients’, *The Guardian*, 21 July 1963, 18.

111 Cartwright, *Patients and their Doctors*, 156; Cartwright and Anderson, 33.

112 The patient’s background is not given. However, though employment for middle-class women was increasing, structural discrimination meant migrant, Black and working-class women were still more likely to require paid work: McCarthy, *Double Lives*, 207–26.

113 Amy Townley, ‘GPs and Patients’, *The Guardian*, 21 July 1963, 18.

114 Peter Bishop, ‘Surveying the “waiting room”’, *Architectural Theory Review*, 18 (2013), 137.

115 Dolly Smith Wilson, ‘A New Look at the Affluent Worker: The Good Working Mother in Post-War Britain’, *Twentieth Century British History*, 17 (2006), 206–29; Richard Hornsey, *The Spiv and the Architect: Un**ruly Life in Post-War London* (Minneapolis, 2010); M. Shapira, *The War Inside: Psychoanalysis, Total War, and the Making of the Democratic Self in Postwar Britain* (Cambridge, 2013). Such projects were also racialized: Fiona Williams, ‘Race/Ethnicity, Gender and Class in Welfare States: A Framework for Comparative Analysis’, *Social Policies*, 2 (1995), 127–59.

116 ‘Doctors Do Care, Say the Patients’, *Daily Mail*, 16 March 1982, 13.

117 As for hospital antenatal clinics: Davis, *Modern Motherhood*, 90–91.

118 Bevan and Draper, *Appointment Systems in General Practice*, 92, 96, 125.

119 Moran, ‘Queuing up in Post-war Britain’, 297–8.

120 Moran, ‘Queuing up in Post-war Britain’, 293–4, 297–300.

121 Moran, ‘Queuing up in Post-war Britain’, 300–1.

122 Moran, ‘Queuing up in Post-war Britain’, 295–6.

123 Medical World, *Appointment Systems in General Practice*, 13.

124 Medical World, *Appointment Systems in General Practice*, 4; Central Health Services Council Standing Medical Advisory Committee, *The Field Work of the Family Doctor*, 27.

125 Cartwright and Anderson, *General Practice Revisited*, 30.

126 Cartwright and Anderson, *General Practice Revisited*, 32.

127 ’14 days to see your doctor’.

128 Stimson and Webb, *Going to See the Doctor*, 20–21.

129 Michael Courtenay, ‘The General Practice Dilemma’, *The Lancet*, 20 July 1974, 153.

130 One survey of 400 self-referred patients at the Glasgow Western Infirmary found that 15 per cent attended because ‘the appointment system would mean a delay in seeing the family doctor’: John Roper, ‘Patients with Trivial Complaints Referred Themselves into Hospitals, Report Says’, *The Times*, 29 January 1971, 4.

131 K. Johnstone, ‘Trying your Patients’, *The Guardian*, 1 February 1979, 13.

132 ‘Better Surgeries Urged’, *The Guardian*, 13 January 1970, 18.

133 Linda Christmas, ‘Patients Strong’, *The Guardian*, 7 July 1976, 7.

134 Cartwright and Anderson, *General Practice Revisited*, 32–35; Cunningham, ‘Doctors Avoid “unnecessary” calls’, 7.

135 ‘Better Surgeries Urged’, 18.

136 ‘Organisation in General Practice’, *The Lancet*, 277 (1961), 756; Office of Health Economics, *The Work of Primary Medical Care*, 37.

137 Salisbury, ‘‘Between-time Stories’.

138 Jane Lewis, ‘The Medical Profession and the State: GPs and the GP Contract in the 1960s and 1990s’, *Social Policy and Administration*, 32 (1998), 132–50; Glen O’Hara, *From Dreams to Disillusionment: Economic and Social Planning in 1960s Britain* (Basingstoke, 2007).

139 Alistair Fair, ‘Modernization of our Hospital System’: The National Health Service, the Hospital Plan, and the ‘Harness’ Programme, 1962-77’, *Twentieth Century British History*, 29 (2018), 547–75. Cf: Ed DeVane, ‘Pilgrim’s Progress: The Landscape of the NHS Hospital, 1948-70’, *Twentieth Century British History*, Advanced Access 5 July 2021.

140 Bosanquet and Salisbury, ‘The Practice’, 50–62; Alongside international philanthropy: Andrew Seaton, ‘The Gospel of Wealth and the National Health: The Rockefeller Foundation and Social Medicine in Britain’s NHS, 1945-60’, *Bulletin of the History of Medicine*, 94 (2020), 91–124.

141 John Cunningham, ‘What’s up, Doc?’, *The Guardian*, 8 March 1973, 11.

142 ‘If Single-Handed Doctors Go’, *The Times*, 6 September 1965, 9.

143 Bertha Turner, ‘Appointments Systems in General Practice’, *BMJ* (30 January 1965), 319.

144 M. Courtenay, ‘A Design for General Practice’, *The Lancet*, 9 November 1963, 1005.

145 Chris Waters, ‘“Dark strangers” in our Midst’: Discourses of Race and Nation in Britain, 1947-63’, *Journal of British Studies*, 36 (1997) 207–38.

146 Lawrence, *Me, Me Me*; James Vernon, *Distant Strangers: How Britain Became Modern* (Berkeley, 2014).

147 Betty Thorne, ‘Who Helps Now?’, *The Guardian*, 12 July 1963, 8.

148 Irvine Loudon, ‘The Concept of the Family Doctor’, *Bulletin of the History of Medicine*, 58 (1984), 347–62.

149
*Family Doctor* was published by the British Medical Association, 1951–67; *Doctor Finlay’s Casebook* (BBC TV, 1962–1971); John Berger and Jean Mohr, *A Fortunate Man: The Story of a Country Doctor* (New York, [1967] 1997).

150 Chelsea Saxby, ‘Nostalgia and the NHS’, *The Polyphony*, 7 April 2020. <https://thepolyphony.org/2020/04/07/nostalgia-and-the-nhs/#_ftn1>.

151 Bevan and Draper, *Appointment Systems in General Practice*, 65.

152 ‘Appointment Systems in General Practice’, *The Lancet*, 2 December 1967, 1190.

153 Medical World, *Appointment Systems in General Practice*, 5–6.

154 Elizabeth Langland, ‘Nobody’s Angels: Domestic Ideology and Middle-Class Women in the Victorian Novel’, *PMLA*, 107 (1992), 290–304.

155 Sharing similar gendered tropes around nursing: Sarah Chaney, ‘Before Compassion: Sympathy, Tact and the History of the Ideal Nurse’, *Medical Humanities* (2020): doi: 10.1136/medhum-2019-011842. On GPs and psychosocial perspectives: Shaul Bar-Haim, ‘“The drug doctor”: Michael Balint and the Revival of General Practice in Post-War Britain’, *History Workshop Journal*, 86 (2018), 114–32.

156 ‘“Too bossy” girls upset surgery patients’, *Daily Mail*, 16 July 1973, 3; Jill Tweedie, ‘Middle Class Blues’, *The Guardian*, 2 March 1970, 9.

157 ‘If Single-Handed Doctors Go’.

158 ‘Criticism of Doctors’ Receptionists’, *The Times*, 22 February 1978, 6.

159 Cartwright and Anderson, *General Practice Revisited*, 83; John Cunningham, ‘Patients have a Prescription for the GPs’, *The Guardian*, 16 July 1973, 7.

160 T. H. Marshall, *Citizenship and Social Class and Other Essays* (Cambridge, 1950).

161 Hugh Hebert, ‘Appointments that Bring Disappointment’, *The Guardian*, 22 February 1978, 2; ‘‘Too bossy’ Girls’.

162 Cartwright and Anderson, *General Practice Revisited*, 83, 93; Ann Clwyd, ‘Patients Set Out their Grumbles’, *The Guardian*, 18 October 1976, 5.

163 ‘Doctors Do Care’, 13.

164 de la T. Mallett, ‘An Appointment System in General Practice’, 495.

165 Fry et al., ‘Appointments in General Practice’, 397.

166 ‘Appointment Systems in General Practice’, 1190.

167 Simon Jenkins, ‘A Critical Assessment of Appointment Systems in General Practice’, *The Practitioner*, 199 (1967), 807.

168 ‘Appointment Systems in General Practice’, 1190.

169 Churchill originated the phrase in relation to post-war social security, but it was quickly subsumed into popular culture: Nicholas Timmins, *The Five Giants: A Biography of the Welfare State* (London, 1996), 48.

170 On spatial mobility’s inequalities: Simon Gunn, ‘Spatial Mobility in Later Twentieth Century Britain’, *Contemporary British History*, Advance Access (2021): <https://doi.org/10.1080/13619462.2020.1858060>

171 On the inequitable deputation of waiting to women: Richard Hoggart, *The Uses of Literacy* (London, 1957 [2017]), 25.

172 D. C. Morrell, M. E. Evans, R. W. Morris, and M. O. Roland, ‘The “five minute” Consultation: The Effect of Time Constraint on Clinical Content and Patient Satisfaction’, *BMJ*, 29 March, 1986, 870–3.

173 Gordon Horobin and Jim McIntosh, ‘Time, Risk and Routine in General Practice’, *Sociology of Health and Illness*, 5 (1983), 312–31.

174 This was formalized with the 1966 contract: ‘Introducing the Charter’, *The Lancet*, 8 October 1966, 806.

175 On older practices: Keir Waddington, ‘Unsuitable Cases: The Debate over Outpatient Admissions, the Medical Profession and Late-Victorian London Hospitals’, *Medical History*, 42 (1998), 26–46.

176 Bruce Taylor, ‘Patient Use of a Mixed Appointment System in an Urban Practice’, *BMJ*, 10 November 1984, 1277–8; A. T. Harrison, ‘Appointment Systems: Feasibility Study of a New Approach’, *BMJ*, 6 June 1987, 1465–6.

177 See letters of complaint on file: TNA, London, MH 153/243, ‘Arrangement of Surgery Hours: Appointments Systems’, 1962–65.

178 BL, ‘Margaret Norton’, Part 5 c. 2m 45s.

179 J. Cunningham, ‘Doctors Avoid “Unnecessary” Calls’, *Guardian*, 2 May 1973, 7.

180 BL, Oral History of General Practice, C648/10, ‘Gordon Jenkins interviewed by Michael Bevan’, 1992, Part 3 c. 23m.

